# What an RVU experiment taught me about Medical Physics

**DOI:** 10.1002/acm2.70451

**Published:** 2025-12-29

**Authors:** Timothy D. Solberg

**Affiliations:** ^1^ Department of Radiation Oncology University of Washington Seattle Washington USA

**Keywords:** medical physics workforce, organizational behavior, professional collaboration, resource allocation, staffing models

While my days of leading large, multidisciplinary teams are behind me, I remain acutely aware of the staffing challenges facing medical physics across the United States. Over the course of my career, I've witnessed the full spectrum, from periods in which positions were scarce and competition for jobs was intense to the current landscape, where shortages of qualified physicists strain clinics of every size. These cycles are familiar, and I have no reason to believe they won't recur. The pressures they create—for recruitment, retention, training, and workload distribution—continue to shape the day‐to‐day realities of the field, regardless of whether one is practicing in an academic center or a community clinic.

It's within this context of recurring workforce pressure that conversations about structure, accountability, and resource allocation inevitably arise. On two occasions, I've been asked by health‐system leadership to develop a task‐based, transparent model for physics resource allocation and accountability, drawing on the relative value unit (RVU) model that governs physician work valuation. It is an appealing idea precisely because it promises clarity, fairness, and an evidence‐driven basis for staffing models and workload distribution. It is also inherently challenging, raising complex questions about how to quantify cognitive work, clinical judgment, quality assurance activities, and the nonlinear effort involved in safe radiotherapy delivery. In this editorial, I reflect on the origins and current state of the RVU system, particularly as it may offer guidance—conceptual or cautionary—for developing similar frameworks in medical physics. I also share observations on what has and hasn't worked, as well as the potential pitfalls that any such system must confront if it is to serve clinical practice rather than distort it.

In the late 1980s, the U.S. healthcare system faced a growing crisis: physician payments lacked transparency, fairness, and consistency across specialties. In response, the Resource‐Based Relative Value Scale (RBRVS) was developed, spearheaded by Harvard economist William Hsiao.^1^, ^2^ Shortly thereafter, the Omnibus Budget Reconciliation Act of 1989 authorized the creation of the Medicare Fee Schedule (MFS) and the use of RBRVS for Medicare physician payments. The MFS, which went into effect on January 1, 1992, used RVUs to assign specific payment amounts for each service. The goals of the RVU system were well‐intentioned: to allocate value in proportion to time, effort, skill, and risk, and ultimately, to address the issue of rising medical costs by creating a system that incentivizes efficient and effective care while curbing specialist‐driven cost inflation.

At its inception, the RVU model was a rational attempt to bring structure to physician reimbursement. The physician work component—which includes time, mental effort, technical skill, and stress—was meant to recognize the complexity and labor intensity of different services. Over the ensuing three decades, the RVU system has become calcified into every corner of U.S. healthcare. As it expanded, it enabled the growth of a massive administrative infrastructure within healthcare, gradually reshaping clinical behavior in ways that have become deeply troubling. Consequently, RVU‐centric compensation schemes distort provider behavior by rewarding volume over value, procedures over thinking, and patient throughput over long‐term care.^3^

Additionally, as commercial insurers adopted the system, RVUs morphed from reimbursement tools into tools of surveillance and incentive.^4^ In most health systems, physician compensation is now tethered directly to RVU output, often through productivity‐based bonuses or salary floors. The original stem of reform, a more equitable, evidence‐based pricing system, has fractured under market pressures, yet the RVU itself persists. Its logic may be broken, but its legacy remains, shaping productivity metrics, salary bonuses, and even institutional prestige.

Ironically, the initial goals to bring transparency, fairness, and consistency to physician reimbursement have become a driver of inflationary costs in American healthcare. Volume incentives lead to overutilization. Procedural bias and specialty inflation favor interventions over primary care and preventive services, and specialty societies routinely advocate for increases in the RVU valuation of their procedures. The administrative complexity associated with coding, billing, optimization, compliance, and tracking RVU production contributes to an excessive operational overhead.

Further, while the RVU system is designed to measure and reward clinical productivity, academic environments often signal—both explicitly and implicitly—that time spent in clinical care is less valuable than time spent in meetings, writing grants, fulfilling accreditation tasks, mentoring trainees, or serving on committees. Faculty who take on these roles are often seen as “team players” and rewarded with visibility, leadership opportunities, or promotion—yet these tasks are rarely tracked, funded, or protected under RVU‐based compensation models. The unintended consequence is a culture in which clinicians may be structurally and reputationally incentivized not to see patients. Seeing fewer patients can mean fewer RVUs—but also fewer scheduling headaches, fewer clinical risks, and more freedom to participate in the academic prestige economy. Over time, this dynamic distorts both workload and morale, as departments struggle to maintain clinical access while over‐rewarding administrative participation. Without a deliberate effort to quantify and balance these competing demands, the RVU framework becomes not only inadequate—it becomes corrosive.

Establishing an RVU‐like system for medical physics is associated with all of the drawbacks described above, with some additional unique challenges. First, there is no centralized physics RVU authority, as there is with physician services through the AMA/Specialty Society RVS Update Committee (RUC) and CMS. Without such an adjudicating body, it becomes extremely difficult to establish and maintain a standardized baseline of effort across diverse tasks, even within an individual institution. Physics work is heterogeneous—ranging from direct patient support, such as treatment planning and special procedures, to quality assurance, equipment calibration, radiation safety, research, and education. Physics effort is also inherently variable—by modality (linac, Gamma Knife, proton therapy, brachytherapy), by institutional resources, by case mix, and by expectations—and much of our work is preventive, reducing risk rather than generating billable events. Attempts to impose rigid values on such heterogeneous work inevitably oversimplify tasks and encourage “checkbox” behavior, where individuals chase widgets, countable surrogates of effort, rather than focusing on patient safety, innovation, problem‐solving, or active intellectual participation. Unlike medical or surgical procedures, these activities do not map neatly onto discrete CPT codes or work units. Rather, the value is less tangible, in part because much of the work is preventive in nature, reducing risk rather than generating checked boxes.

I experienced this firsthand. We developed an RVU‐like system through a careful design process that drew on appropriate benchmark references, including the Abt reports, ASTRO and IAEA guidelines, and others,^5^, ^6^, ^7^, ^8^, ^9^, ^10^ and with input and open discussion from the entire division. We piloted the system, solicited feedback, and adjusted work values accordingly. Within 6 months of implementation, it was clear that we had failed; the process unraveled for all the reasons outlined above. The moment RVU‐credits became a currency, people grew protective of their own tasks, increasingly vocal about perceived inequities, and more focused on documenting effort than contributing meaningfully. Comparisons between colleagues escalated, minor grievances expanded, and the instinct to collaborate gave way to the instinct to defend one's numbers. Work falling outside the defined widgets of effort quickly felt like uncompensated labor. We introduced a moral hazard: individuals were incentivized to optimize their own measurable output, even when doing so conflicts with the collective good. As one colleague aptly remarked, “We've lost normal human being interaction.” In a safety‐critical field like medical physics, this is not a theoretical concern; it is a structural risk.

In retrospect, the outcome could have been predicted; the true value of medical physics lies not in counting widgets, tallying hours, or comparing one's output to that of colleagues, but in safeguarding patients through collaboration, judgment, and shared responsibility. The work we do is fundamentally relational and situational: risk is reduced not by individual productivity metrics but by collective awareness, shared expertise, and the continuous exchange of information that keeps small problems from becoming large ones. No spreadsheet can substitute for the informal conversations, quick questions, and unplanned insights that occur when professionals trust one another and work side by side.

It is tempting to believe that more sophisticated tools, automated tracking systems, AI‐derived effort estimates, or increasingly granular task logs might succeed where earlier RVU‐like models failed. My experience suggests otherwise. Improving the precision of measurement does not resolve the underlying problem; it often accelerates it by further displacing trust, judgment, and shared responsibility.

In the end, we replaced the RVU‐like framework with something far simpler—physicists sitting together each day in the clinic, addressing tasks and tackling problems collaboratively. This approach not only served the clinic more effectively but also fostered cross‐training, strengthened teamwork, and restored the face‐to‐face interaction that builds trust and sustains collective vigilance. Instead of optimizing for individual output, we optimized for situational awareness, shared accountability, and a culture where everyone felt responsible for the whole rather than a slice of it.

Looking back, the lesson is not that structure is unnecessary, but that the wrong kind of structure can quietly erode the very qualities that make a high‐stakes clinical environment safe. Medical physics will always need clarity, consistency, and equitable workload distribution. What it cannot afford is a model that reduces professional judgment to widgets or substitutes metrics for meaningful engagement. Whatever frameworks emerge in the future—whether for staffing, valuation, or workload—they must begin with an honest recognition that our greatest strength is not individual productivity, but the collective vigilance that arises when skilled professionals work together, openly, in service of patients who depend on us.

## AUTHOR CONTRIBUTIONS

Timothy Solberg was responsible for conceptualization, content, investigation, writing, reviewing, and editing.

## CONFLICT OF INTEREST STATEMENT

The author declares no conflicts of interest.

## References

[1] Hsiao WC , Braun P , Becker ER , Thomas SR . The resource‐based relative value scale. toward the development of an alternative physician payment system. JAMA. 1987;258:799‐802. doi:10.1001/jama.1987.03400060075033 3613008

[2] Hsiao WC , Braun P , Yetema D , Becker ER . Estimating physicians work for a resource‐based relative‐value scale. NEJM. 1988;319:835‐841. doi:10.1056/NEJM198809293191305 3412414 10.1056/NEJM198809293191305

[3] Emanuel EJ , Fuchs VR . The perfect storm of overutilization. JAMA. 2008;299(23):2789‐2791. doi:10.1001/jama.299.23.2789 18560006 10.1001/jama.299.23.2789

[4] Berenson RA , Goodson JD . Finding value in unexpected places—fixing the medicare physician fee schedule. NEJM. 2016;374(14):1306‐1309. doi:10.1056/NEJMp1600999 26959109 10.1056/NEJMp1600999

[5] Mills MD . Analysis and practical use: the Abt Study of Medical Physicist Work Values for Radiation Oncology Physics Services–round II. J Am Coll Radiol. 2005;2(9):782‐789. doi:10.1016/j.jacr.2005.02.009 17411927 10.1016/j.jacr.2005.02.009

[6] Klein EE . A grid to facilitate physics staffing justification. J Appl Clin Med Phys. 2009;11(1):263‐273. doi:10.1120/jacmp.v11i1.2987 10.1120/jacmp.v11i1.2987 PMC5719772 20160679

[7] Battista JJ , Clark BG , Patterson MS , et al. Medical physics staffing for radiation oncology: a decade of experience in Ontario. Canada J Appl Clin Med Phys. 2012;13(1):93‐110. doi:10.1120/jacmp.v13i1.3704 10.1120/jacmp.v13i1.3704 PMC5716143 22231223

[8] Safety is no accident: a framework for quality radiation oncology care, Am Soc Rad Oncol, 2012 (updated 2019). https://www.astro.org/practice‐support/quality‐and‐safety/safety‐is‐no‐accident

[9] Abt Associates . The abt study of medical physicist work values for radiation oncology physics services: round IV. final report. Am Assoc Physicists Med. 2015. https://www.aapm.org/pubs/reports/ABTIVReport.pdf

[10] International Atomic Energy Agency . Staffing in Radiotherapy: an Activity Based Approach: Human Health Reports No. 13. IAEA; 2015. https://www.iaea.org/publications/10800/staffing‐in‐radiotherapy‐an‐activity‐based‐approach

